# Comparison of Metal-Backed Free-Space and Open-Ended Coaxial Probe Techniques for the Dielectric Characterization of Aeronautical Composites ^†^

**DOI:** 10.3390/s16070967

**Published:** 2016-06-24

**Authors:** Patricia López-Rodríguez, David Escot-Bocanegra, David Poyatos-Martínez, Frank Weinmann

**Affiliations:** 1 Radiofrequency Area, National Institute for Aerospace Technology (INTA), Torrejón de Ardoz, 28850 Madrid, Spain; escotbd@inta.es (D.E.-B.); poyatosmd@inta.es (D.P.-M.); 2 Department Antenna Technology and Electromagnetic Modelling AEM, Fraunhofer Institute for High Frequency Physics and Radar Techniques (FHR), 53343 Wachtberg, Germany; frank.weinmann@fhr.fraunhofer.de

**Keywords:** antenna probes, permittivity, characterization, free-space, composite materials, fiberglass, coaxial probe, split cavity resonator

## Abstract

The trend in the last few decades is that current unmanned aerial vehicles are completely made of composite materials rather than metallic, such as carbon-fiber or fiberglass composites. From the electromagnetic point of view, this fact forces engineers and scientists to assess how these materials may affect their radar response or their electronics in terms of electromagnetic compatibility. In order to evaluate this, electromagnetic characterization of different composite materials has become a need. Several techniques exist to perform this characterization, all of them based on the utilization of different sensors for measuring different parameters. In this paper, an implementation of the metal-backed free-space technique, based on the employment of antenna probes, is utilized for the characterization of composite materials that belong to an actual drone. Their extracted properties are compared with those given by a commercial solution, an open-ended coaxial probe (OECP). The discrepancies found between both techniques along with a further evaluation of the methodologies, including measurements with a split-cavity resonator, conclude that the implemented free-space technique provides more reliable results for this kind of composites than the OECP technique.

## 1. Introduction

The study of the electromagnetic properties of composites has attracted much attention in the last decades with the aim of developing materials with an expected behaviour. Considering that the properties of a composite material are related to the properties and the amount of its constituents, the trend is to develop materials with customized characteristics to be used for certain purposes. However, their prediction out of the composite constituents is a long-standing problem, which has not yet been completely solved, and, therefore, interest in experimentally characterizing composite materials is still growing [1]. In fact, along the years, numerous studies have been dedicated to the electromagnetic characterization of materials and the acquired knowledge has been utilized in many industrial fields such as defense, medical, automotive or engineering [2,3].

Recently, the use of composite materials in the construction of aerospace structures has increased dramatically in such a way that certain aircraft parts like fairings, spoilers, radomes, propellers or flight control surfaces are now entirely made of composites [4,5,6]. In addition, some new generation fuselages and wings are designed to be almost fully composite providing lighter and more fuel-efficient aircraft, like current unmanned aerial vehicles (UAVs).

Depending on the final application of these materials within an aeronautic structure, their electromagnetic characterization is desired for different purposes: radar-cross section, electromagnetic absorption, radome effects in radiation and scattering patterns, conductivity, shielding, etc. Additionally, although aircraft certification is performed by experimental testing, computational tools are currently being considered for future certification purposes [7]; therefore, numerical solvers should also have information about the electromagnetic behaviour of the constitutive materials of the aircraft under test.

To extract the dielectric properties of a material, several experimental methods exist. However, the most suitable method for a certain specimen mainly depends upon the specific frequency band of use, its expected losses, the required accuracy and on the nature and shape and size of the sample [8,9]. Resonant methods, like cavity resonators, are generally applied to low-loss material characterization; however, they only return information about the dielectric properties of a material in a discrete number of frequencies. These methods measure the resonance frequency and quality factor variation of a cavity and some of them require a meticulous sample preparation before measurements take place. On the other hand, non resonant methods, including open-ended coaxial probe, transmission line technique and free-space, sense the reflected and/or transmitted signal through a specimen material to characterize it in a broad frequency band [9,10,11] and, except for the transmission line method, they are less restrictive in sample preparation compared to resonant methods.

The aim of this paper is to present such characterizations for several samples of composite materials that take part in an actual UAV fuselage utilized by the Spanish National Institute for Aerospace Technology (INTA), called SCRAB-II (manufactured by Sistemas de Control Remoto (SCR), Spain) [12]. The available sample materials for this research comprise a number of flat sheets of foam core fiberglass sandwich with expected low losses, and the frequency range of interest comprises from 8 to 16 GHz. Glass-fiber composites are widely used in applications in which transparency to electromagnetic radiation is required, including radomes and aerial covers. With the advent of new lighter aerial platforms, the introduction of these composites into the fuselage is today a reality [4] and, as such, their electromagnetic characterization is of high interest.

## 2. Materials and Methods

### 2.1. Materials under Test

Material characterization in electromagnetics refers to the macroscopic behaviour of materials when subjected to an external electromagnetic field. According to Maxwell’s equations, the response of a material to an electromagnetic field is determined by three constitutive parameters, namely permittivity (*ϵ*), permeability (*μ*) and conductivity (*σ*) (which, for low-conductivity materials, is related to the permittivity). These parameters determine to which extent the field affects the behaviour of the material at a given frequency, and, therefore, they are not constant; indeed, they vary with frequency, temperature, orientation and molecular structure of the material [1,13].

Relative permittivity, $\epsilon_{r}$, describes the interaction of a material with an electric field; it is a dimensionless complex quantity defined as

(1) $$\epsilon_{r}=\frac{\epsilon }{\epsilon_{0}}=\epsilon_{r}^{\prime }-j\epsilon_{r}^{\prime \prime }=\epsilon_{r}^{\prime }\left(1-j\tan\delta \right)$$

where $\epsilon_{0}=8.854\times 10^{-12}$ F/m is the permittivity of free space, *ϵ* is the complex permittivity in F/m and $\tan\delta =\epsilon_{r}^{\prime \prime }/\epsilon_{r}^{\prime }$ is called the loss tangent.

Similarly, the relative permeability of a material, $\mu_{r}=\mu /\mu_{0}=\mu_{r}^{\prime }-j\mu_{r}^{\prime \prime }$, relates to its response under an interaction with a magnetic field and is equal to unity for non-magnetic materials. Just like permittivity, this quantity is expressed related to the permeability of free space $\mu_{0}=4\pi \times 10^{-7}$ N/A${}^{2}$. For simplicity, the terms "relative" are usually omitted. As a result of an electromagnetic field interaction, materials experience energy storage and energy dissipation. Energy storage describes the exchange of energy between the field and the material and is determined by the real part of the permittivity and permeability, while the energy dissipation refers to the energy absorbed by the material, which is represented by the imaginary parts of both parameters [1,9].

**Figure 1:**
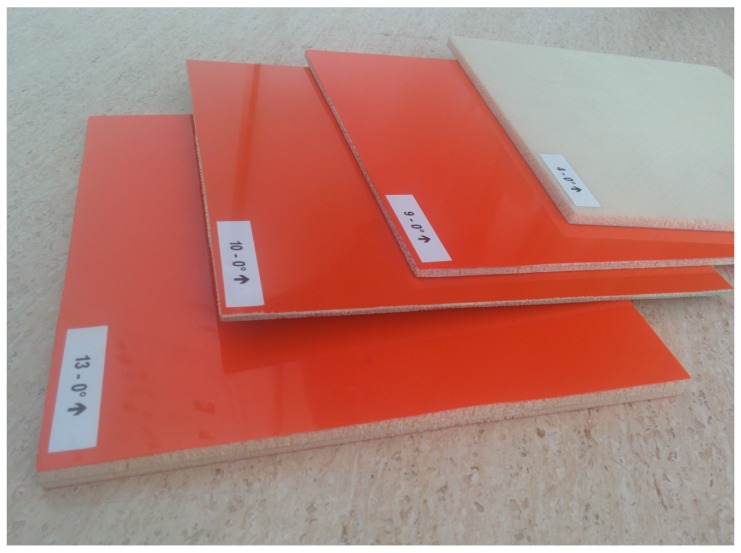
SCRAB-II materials under test.

In this study, two sets of materials (a yellow and a white set, depending on the color of their sticker) are available. While the white set remains at INTA premises for different tests, the yellow set is frequently shared with other institutions. Each set consists of a number of 4 flat parallel faced samples of foam core fiberglass sandwich composite of dimensions 200 × 200 mm utilized in SCRAB-II. Note that fiberglass is non-magnetic ($\mu_{r}=1$) and does not conduct electricity, therefore, only the complex permittivity will be characterized and its expected imaginary part will be very low. Each sample has its own thickness and a different number of composite layers as can be seen in Figure 1 and Table 1. Additionally, two samples of materials with known dielectric properties are also available, Teflon (DuPont, Wilmington, DE, USA) and Eccostock HiK500F (Emerson & Cuming Microwave Products, Randolph, MA, USA) (with $\epsilon_{r}^{\prime }=2.1$ and $\epsilon_{r}^{\prime }=30$, respectively), which will be used as confirmation of results.

### 2.2. Metal-Backed Free-Space Methodology

Free-space methods are non-destructive, and they are based on the extraction of the electromagnetic properties of materials from free-space reflection or transmission measurements [14]. In the present study, free-space techniques are preferred since composite materials can be inhomogeneous along their structure, and this can cause inaccuracies when measuring them in hollow metallic waveguides (note that, in this last case, unwanted higher-order modes may be excited at the air-dielectric interface [1]). Furthermore, the available samples have a specific shape (parallel flat plates) and dimensions that are appropriate for free-space and need not be further machined to fit a specific fixture. Lastly, free-space methods are adequate to characterize materials in a broad frequency range in a non-contact and non-destructive manner.

INTA owns an anechoic chamber facility (BIANCHA) suitable for free-space measurements [15]. BIANCHA consists of a dual-axis azimuth turntable and two antenna probes situated in two elevated scanning arms, which establish a bistatic, spherical field scanner. These arms can be located at any point of an imaginary hemisphere, hence being able to measure the reflection coefficient at normal incidence or at any given angle. Although this facility may perform improved measurements of material absorption and characterization with the option for bistatic measurements, in the present research, since the materials utilized along this study are non-magnetic, the measurement of the reflected energy in a monostatic configuration is enough to infer $\epsilon_{r}$.

**Figure 2:**
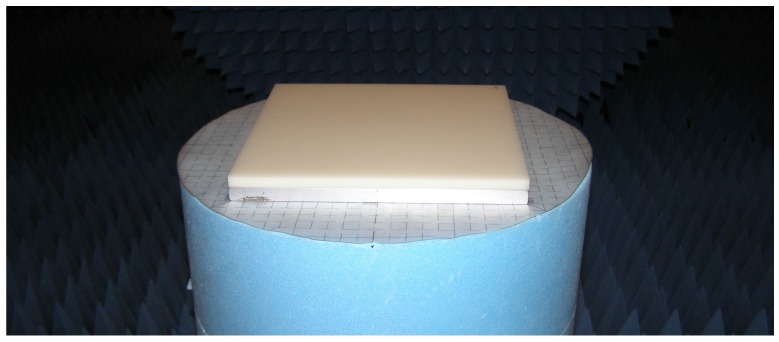
Metal-backed sample of Teflon.

The measurement procedure for extracting the dielectric constants of the materials under test is discussed in the report by Ghodgaonkar et al. [14] and has been used in a variety of research papers [16,17]. Basically, this procedure involves measuring the reflected signal from a flat material sample that has a perfectly conducting plate underneath, just as Figure 2 shows, where the reference material, a 10 mm thick slab of Teflon, is placed over a metal plate.

**Figure 3:**
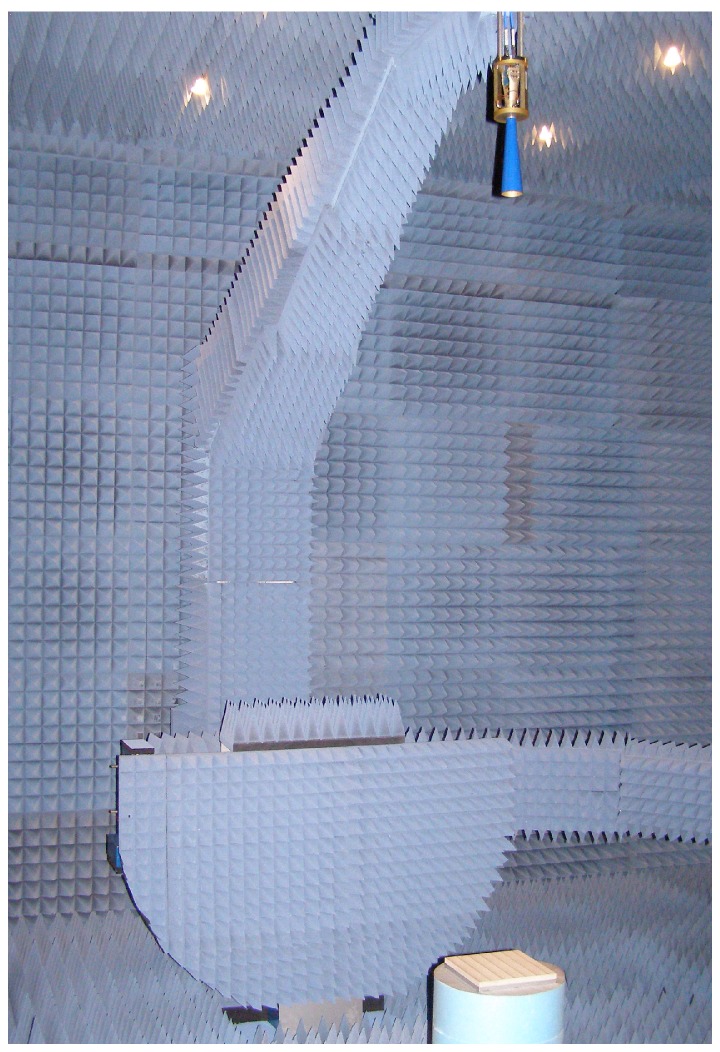
Metal-backed free-space setup.

As the measurement setup in Figure 3 shows, the metal-backed material is placed facing the transmitting antenna at its focus, while the metal plate will act as a perfectly conducting plane (a short). A vector network analyzer (VNA) (a four-port ZVA50 manufactured by Rohde & Schwarz (Munich, Germany)) is utilized to acquire the $S_{11}$ parameter, that is, the reflected energy of the sample-metal specimen. The test setup calibration consists firstly of a full-port calibration of the VNA and then, a calibration with respect to a 200 × 200 mm metal plate that provides a baseline for perfect reflection. Additionally, in order to minimize the diffraction effects and the multiple reflections, background subtraction and software gating in the time domain are applied [18]. Lastly, and with little processing, the reflection coefficient (Γ) is obtained.

From transmission line theory, one can relate the measured reflection coefficient with the normalized input impedance of the sample-metal geometry ($Z_{sc}$), which is directly related to the complex permittivity by Equation (2), where normal illumination is considered and *d* represents the thickness of the material sample:

(2) $$\Gamma =\frac{Z_{sc}-1}{Z_{sc}+1}=\frac{\sqrt{\frac{1}{\epsilon_{r}}}\tanh\left(j\frac{2\pi }{\lambda }d\sqrt{\epsilon_{r}}\right)-1}{\sqrt{\frac{1}{\epsilon_{r}}}\tanh\left(j\frac{2\pi }{\lambda }d\sqrt{\epsilon_{r}}\right)+1}$$

Finding a solution for $\epsilon_{r}$ is not straightforward and the typical approach makes use of root-finding or optimization algorithms to find zeros of the error function presented in Equation (3); that is, the method consists of finding the optimal value of $\epsilon_{r}$ that minimizes the difference between the measured reflection coefficient ($\Gamma_{ref}\left(\epsilon_{r}\right)$) and the theoretically estimated one ($\Gamma_{est}\left(\epsilon_{r}\right)$):

(3) $$f\left(\epsilon_{r}\right)=\left|\Gamma_{est}\left(\epsilon_{r}\right)-\Gamma_{ref}\left(\epsilon_{r}\right)\right|$$

Several root-finding algorithms have been utilized along the years to infer $\epsilon_{r}$ in these type of measurements; the Newton–Raphson and Müller algorithm with deflation are the most popular [14,17]; however, they need an initial good estimate of the permittivity for their convergence. In this research, the estimation of $\epsilon_{r}$ is not known a priori; therefore, finding a solution with these algorithms may be a tedious task. Thus, due to its ability to search within a large solution space, the Particle Swarm Optimization (PSO) algorithm is chosen. PSO was developed by Kennedy and Eberhart [19], and is based on the movement and intelligence of swarms. In this algorithm, a number of *M* particles forming a swarm are initially randomly located in an *N*-dimensional space with an initial random velocity in each dimension. In each iteration, each particle moves over the solution space, remembering the location of its best solution ($p_{best}$) and the location of the other particles’ best solution ($g_{best}$) in the *N*-dimensional space. Its basic operation can be summarized as follows:

1. Initialize a population array of *M* particles with random positions and velocities in the *N*-dimensional space.
2. For each particle, evaluate the fitness function and update its local and global best solution in the *N*-dimensional space ($p_{best}^{n}$ and $g_{best}^{n}$ respectively, being n=1,…,N).
3. For each dimension (n=1,…,N), update the velocity of each particle according to the relative locations of $p_{best}^{n}$ and $g_{best}^{n}$ following Equation (4)
  (4) $$v_{k+1}^{n}=w\cdot v_{k}^{n}+c_{1}\cdot r_{1}\left(p_{best,k}^{n}-x_{k}^{n}\right)+c_{2}\cdot r_{2}\left(g_{best,k}^{n}-x_{k}^{n}\right)$$
  where subindex *k* denotes an iteration, $v_{k}^{n}$ is the velocity of the particle associated to dimension *n* and iteration *k*, $x_{k}^{n}$ the particle location in dimension *n* for iteration *k*, $c_{1}$ and $c_{2}$ are scaling factors, such that $c_{1}$ determines the influence of the local best location on the velocity, and $c_{2}$ determines how much a particle is influenced by the rest; $r_{1}$ and $r_{2}$ are random numbers between 0.0 and 1.0, and *w* is the inertial weight (0.0<w<1.0) that is introduced to determine to what extent each particle remains along its original course.
4. Update particle location $x_{k}^{n}$ on the *N*-dimensional space according to Equation (5), where n=1,…,N:
  (5) $$x_{k+1}^{n}=x_{k}^{n}+v_{k+1}^{n}$$
5. Repeat the process from step 2 until the termination criteria is met.

The PSO has been implemented in Matlab such that, in this case, the number of particles utilized to find an optimum solution is M=30 and the dimensionality of the solution space is N=2, since both the real part and the imaginary part of the permittivity must be estimated. This means that $x_{k}^{1}$ will correspond to $\epsilon_{r}^{\prime }$, while $x_{k}^{2}$ to $\epsilon_{r}^{\prime \prime }$. The two-dimensional solution space is defined between 1 and 35 for dimension 1, and 0 and 5 for dimension 2, that is $\epsilon_{r}^{\prime }$ can take values up to 35, while the maximum value $\epsilon_{r}^{\prime \prime }$ can reach is 5. The fitness function that evaluates the goodness of the solution is defined as Equation (3), where in each iteration $\epsilon_{r}=x_{k}^{1}-jx_{k}^{2}$. The termination criteria is set to a maximum number of 200 iterations; the scaling factors are $c_{1}=c_{2}=2$ as it is proposed in [20], and the inertial weight (*w*) is linearly decreased from 0.9 to 0.1. Note that the maximum velocity ($V_{max}^{n}$) of each particle is set equal to the dynamic range of its respective dimension *n*. Additionally, particles wandering outside the solution space are not evaluated for fitness (invisible wall boundary condition).

As seen, although some parameters exist that should be initialized, they are not as restrictive as those for the Müller or Newton algorithms. Actually, when these algorithms converge, they do it to the same value as the one returned by PSO. Even though the computation time is degraded with PSO in comparison with others, the probability of a proper estimation of the permittivity is raised.

### 2.3. Open-Ended Coaxial Probe

**Figure 4:**
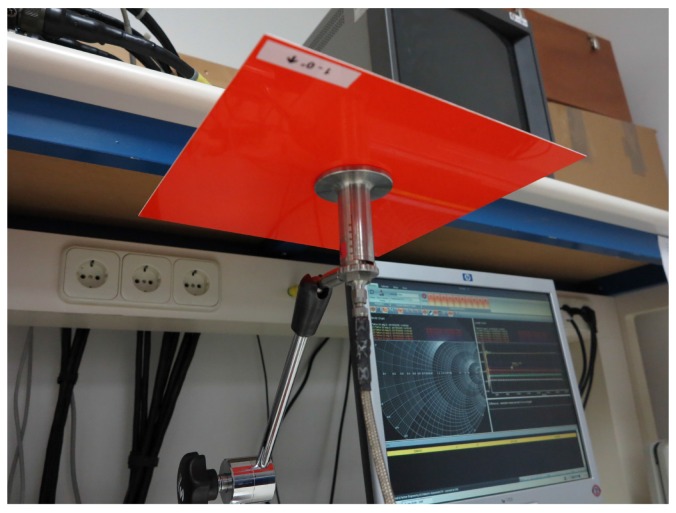
Open-ended coaxial probe setup.

To further evaluate the results obtained with the free-space methodology, the available samples have also been measured with a commercial kit (DAK from SPEAG [21]), which includes an open-ended coaxial probe (OECP) and a commercial software that automatically estimates $\epsilon_{r}$. The OECP is a cut-off section of a coaxial line with its outer conductor extending radially outward, where the dielectric parameters of a material are obtained by measuring the reflected energy. For accurate probe measurements, the probe must be in intimate contact with the material so, in order to avoid air gaps, the material is placed over the OECP just like the setup in Figure 4 shows. Although this technique may be more adequate for liquids or semi-solids, it can be also used for measuring homogeneous flat surface solid materials that have a semi-infinite thickness [3,8]. Therefore, to follow these restrictions, the materials are assumed to be homogeneous along their structure and a metal plate is placed behind each sample (a perfectly conducting plane) to check that the solution is not altered, and so the material can be considered as semi-infinite.

## 3. Results and Discussion

**Figure 5:**
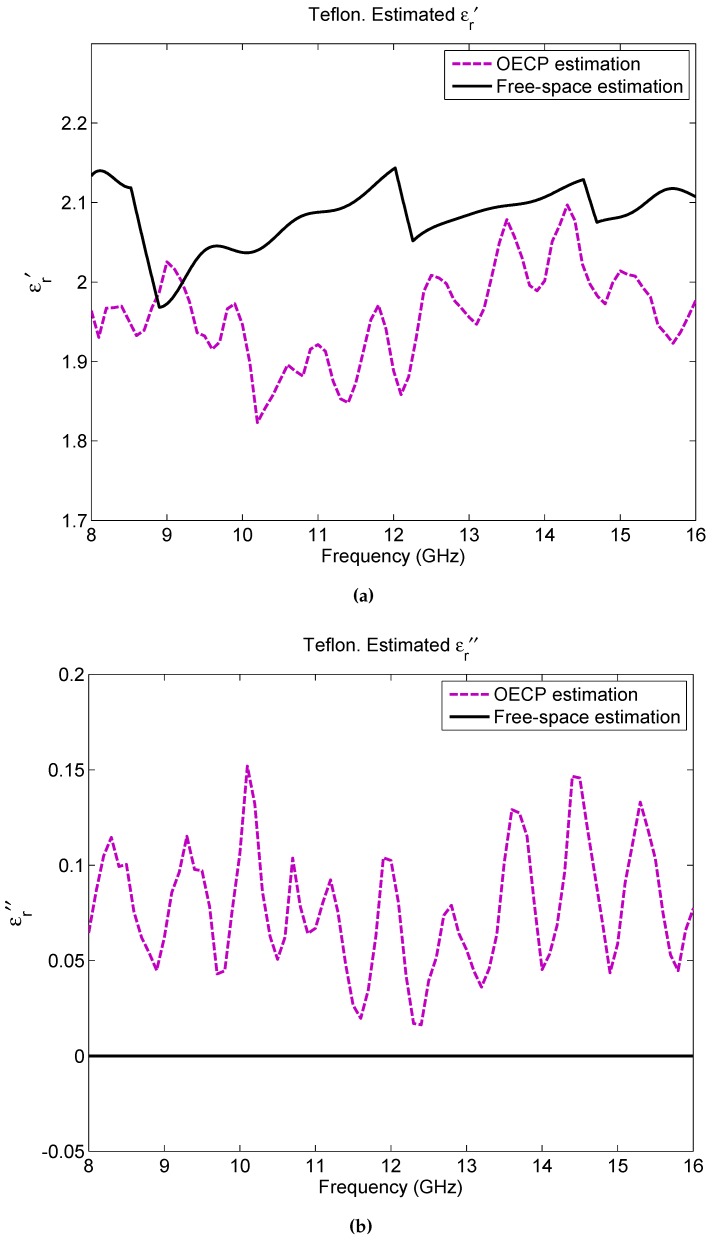
Teflon estimated permittivity: (**a**) $\epsilon_{r}^{\prime }$, (**b**) $\epsilon_{r}^{\prime \prime }$.

Firstly, in order to check the validity of the metal-backed free-space methodology for fiberglass composites, the reference materials are measured with both approaches, free-space and the commercial solution (OECP). Figure 5 depicts the extracted complex permittivity for a 10 mm thick slab of Teflon in the desired frequency range (8–16 GHz) for the two methodologies. As seen, regarding the real part of the permittivity, there exists good agreement between its theoretic value (∼2.1) [13] and its estimation from both the free-space and the OECP measurements. Therefore, since both methodologies coincide, the metal-backed free-space method along with the extraction of the permittivity via the PSO can be considered as a proper approach for the problem faced in this study. In the case of the imaginary part of the permittivity ($\epsilon_{r}^{\prime \prime }$), the results extracted with PSO from the free-space measurement return a mean value of 0, while OECP returns values between 0 and 0.15; it must be noted that $\epsilon_{r}^{\prime \prime }$ is highly sensitive to measurement frequency and experimental errors and when the expected losses are very small, as here, it is difficult to obtain a very accurate value with these two methods. Actually, according to Arthur von Hippel [13], Teflon should have a tanδ for the frequency range studied here of an order of $1\times 10^{-4}$ and, as seen, the values given by OECP do not fulfill this requirement. On the other hand, the values of free-space do not have enough precision to infer such a small tanδ. Therefore, and since the rest of the studied materials are low-loss, more attention will be paid to the real part of the permittivity and only the mean values of the obtained $\epsilon_{r}^{\prime \prime }$ for the whole frequency range will be provided.

**Figure 6:**
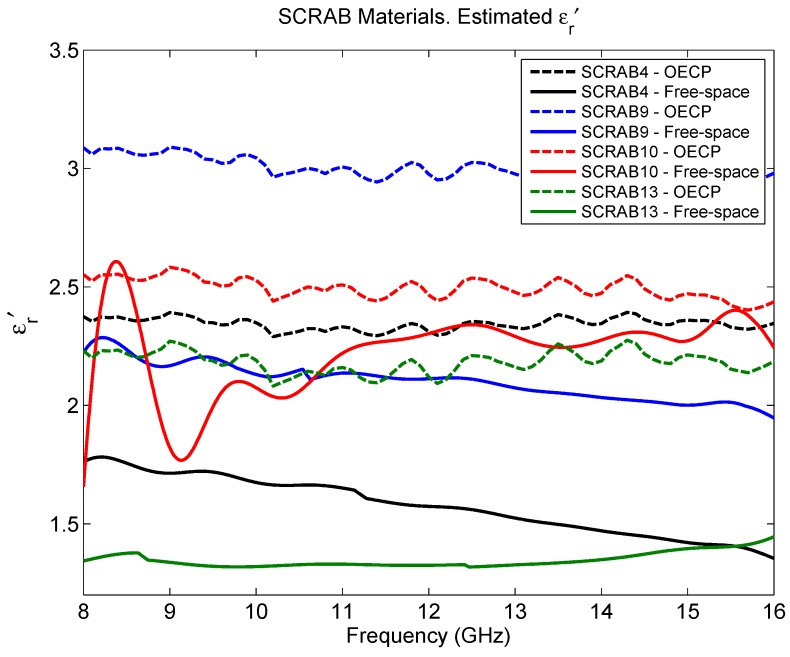
SCRAB-II materials estimated permittivity: $\epsilon_{r}^{\prime }$.

The estimated complex permittivity for the four slabs of fiberglass material corresponding to the white set can be seen in Figure 6, where the results given by OECP and metal-backed free-space are compared. In Table 2, the mean permittivity along the frequency range for each material is summarized together with their thickness. As Figure 6 shows, the real part of the permittivity presents discrepancies between the values given by OECP and those estimated from the measurements in free-space. Only the SCRAB 10 material, the thinnest and most compact among the four samples, has offered similar results with both techniques, but in the rest of the cases, OECP returns higher values of $\epsilon_{r}^{\prime }$ than free-space. Regarding $\epsilon_{r}^{\prime \prime }$, as Table 2 shows, the results obtained with OECP are always higher than what is obtained with free-space. In any case, after the evaluation of Teflon, it was seen that, for low-loss materials, the accuracy achieved with the free-space technique is not enough to infer a proper $\epsilon_{r}^{\prime \prime }$, while the OECP provides a biased result where the obtained $\epsilon_{r}^{\prime \prime }$ is exaggerated.

**Figure 7:**
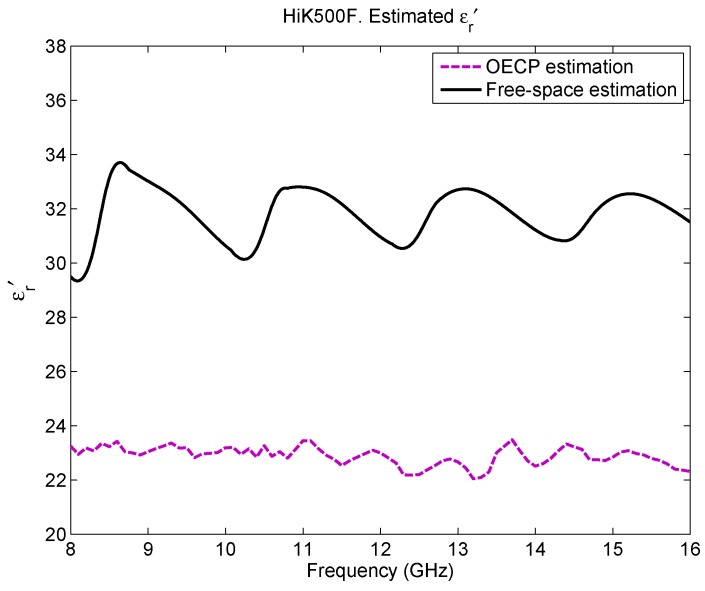
Eccostock HiK500F estimated permittivity: $\epsilon_{r}^{\prime }$.

At this point, in order to decide which solution is more proximate to reality, may it be the one from OECP or the one from free-space measurements, the second reference material is evaluated, a 12.7 mm thick slab sample of Eccostock HiK500F with known $\epsilon_{r}^{\prime }$. Figure 7 depicts its obtained permittivity from both methodologies under study. This material has a known $\epsilon_{r}^{\prime }=30\pm 10\%$ and low losses (expected tanδ<0.002 up to 10 GHz) [22]; therefore, the estimated imaginary part of the permittivity will experience the same behaviour as in the previous cases (free-space does not provide enough resolution to obtain an accurate $\epsilon^{\prime \prime }$ while OECP returns an elevated result). As seen in the figure and in Table 3, there is a big difference between the estimated permittivity with the OECP and with the free-space approach, just like what happened with the materials under test. According to OECP, the real part of the permittivity has a mean value of 22.89, and, hence, it does not fulfill the requirements of the manufacturer; however, the free-space measurement returns a mean value of 31.83, which does comply with the specifications. On the other hand, as Table 3 presents, the extracted mean $\epsilon_{r}^{\prime \prime }$ along the frequency range is very high for both approaches, although free-space gives lower values and, therefore, approximates better to the expected solution.

**Figure 8:**
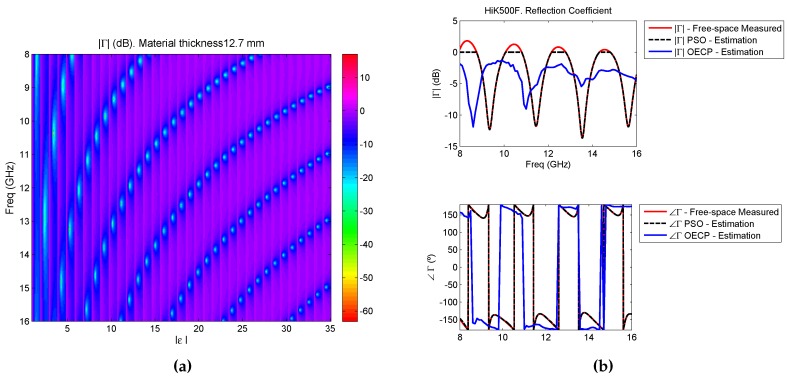
Reflection coefficients: (**a**) theoretic reflection coefficient of a short-circuited material of thickness 12.7 mm according to different values of permittivity (**b**) measured and estimated reflection coefficients for material Eccostock HiK500F, module and phase.

A further study of the measured reflection coefficient gives additional reasons to trust the free-space measurements of certain materials more than the OECP ones. In order to simulate the reflection behaviour of the HiK500F reference material with thickness d = 12.7 mm, the expression of Equation (2) must be followed. Note that this calculation returns the theoretic reflection coefficient of a short circuited sample of a slab material with a specific thickness and permittivity. To do so, the thickness is set to d = 12.7 mm, while the real part of the permittivity has been swept between one and 35 and its imaginary part between 0 and 1.5. The resulting theoretic reflection coefficient is shown in Figure 8a, where the *x*-axis represents the absolute value of the permittivity.

This figure shows the nulls of the magnitude of the reflection coefficient according to the change in frequency and permittivity of a material. On the other hand, Figure 8b shows the measured reflection coefficient in free-space along with the estimated ones using the permittivity retrieved by free-space (using PSO) and by OECP for the complete frequency range for the Eccostock HiK500F reference material. As seen in Figure 8b, the module of the measured reflection coefficient has, in the frequency range of study, four deep valleys where the reflections inside the material cause destructive interference, and these are approximately at 9.5 GHz, 11.5 GHz, 13.5 GHz and 15.5 GHz. Note that these coincide with a phase equal to 180${}^{\circ }$. According to Figure 8a, a material with thickness d = 12.7 mm with a permittivity between 30 and 35 returns a |Γ| with four nulls at around these same frequencies, and, hence, the extracted permittivity should be around these values. The free-space estimation of permittivity by PSO returned a mean value of $\epsilon_{r}=31.83-j0.5$ and as seen in Figure 8b, the estimated reflection coefficient superimposes to the measured one, both module and phase (except in the cases when its module is over 0 dB, where it is truncated since it is due to measurement errors). On the contrary, the one estimated by the OECP and the measured one do not overlap and the nulls of the estimated reflection coefficient are found in the same frequencies that Figure 8a anticipated for a mean value of $\epsilon_{r}=22.89-j2$.

After the evaluation of this reference material, it can be affirmed that, regarding sheets of foam core fiberglass sandwich with expected low losses, metal-backed free-space measurements are more reliable in the dielectric properties’ extraction than OECP. Note that the probe is very sensitive and a bad contact or a small roughness in the surface of the material can corrupt a measurement. Nevertheless, here it was seen that the slab of Teflon provided a similar permittivity for both OECP and free-space; this is mainly due to the fact that the OECP procedure expects a homogeneous and isotropic semi-infinite material and the Teflon sample accomplished these requirements while the SCRAB-II materials did not. Additionally, it should also be noticed that OECP performs the measurements in an exact point of the material surface where the inner structure might slightly differ from another point. Therefore, should the probe not be able to penetrate all the different composite layers or find inhomogeneities in the sandwiched foam, the retrieved permittivity might deviate from its real one. On the other hand, the free-space method is based on the reflection of the whole structure, material-metal, and, as a result, all the layers of the composite material along with the air bubbles of the foam are sensed. Therefore, the obtained permittivity should be closer to reality.

**Figure 9:**
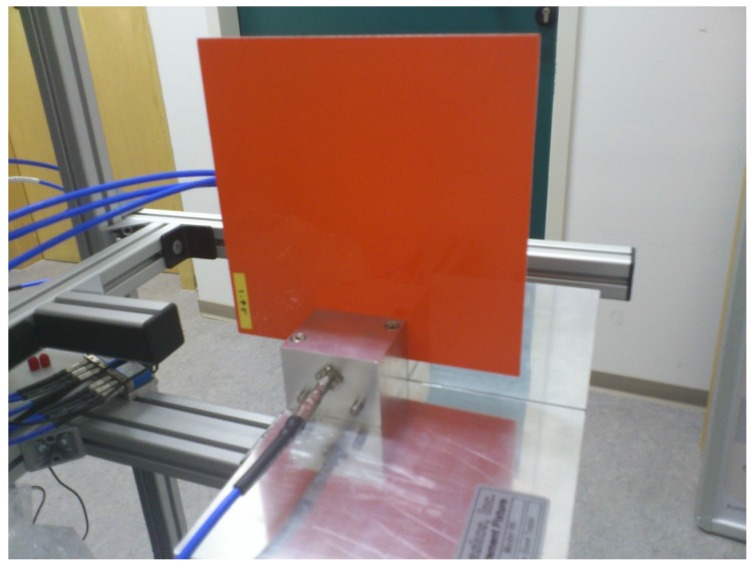
Measurement of a SCRAB-II slab material in a split cavity resonator.

**Figure 10:**
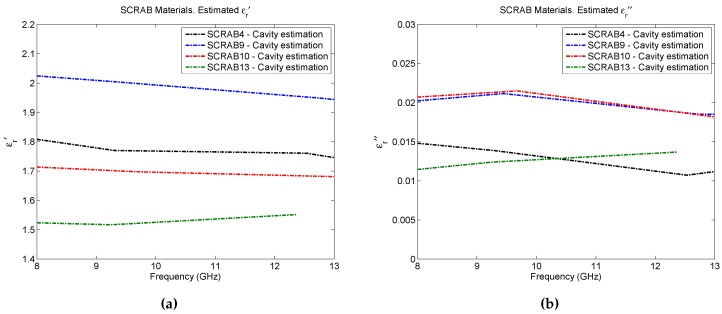
SCRAB-II materials estimated permittivity with split cavity resonator: (**a**) $\epsilon_{r}^{\prime }$ (**b**) $\epsilon_{r}^{\prime \prime }$.

Lastly, and as a final verification of results, the yellow set of the SCRAB-II materials under test have been measured with a commercial split cavity resonator at the Fraunhofer Institute for High Frequency Physics and Radar Techniques (FHR) premises in Wachtberg, Germany. The instrument is a rectangular split resonator, model 015 developed by Damaskos Inc. (Concordville, PA, USA) [23] consisting of two cavity resonators supported by a software package which performs the control of the VNA and the data processing needed to extract the complex permittivity. Recall that resonant cavities are structures with a high quality factor (Q) that resonate at discrete frequencies. When a material is inserted into the cavity, it affects its resonant frequency and quality factor such that from their variation, the complex permittivity of the material can be calculated at specific frequencies. The measurement procedure is the same as the one explained in [24] where a special mounting and measurement device with solid guidance structure is employed for the assembly of the materials under test inside the cavity. Figure 9 presents the measurement setup. Note that the measurements of these materials with this cavity only provide stable results up to 13 GHz due to the difficulties found in the proper determination of the resonant frequencies at higher frequencies. Therefore, only results in a frequency range from 8 to 13 GHz are depicted in Figure 10. Note also that, for the SCRAB 13 material, only information up to 12.5 GHz is available. For a more reliable comparison, the mean values of both the real and the imaginary part of the permittivity estimated with the three techniques for this frequency range are shown in Table 4. As seen in the table, concerning the real part of the permittivity, the mean values retrieved by the cavity are closer to the estimations of free-space, and they have a variation of around ±10%, except for SCRAB 10. On the other hand, the OECP results are always higher. Remember that free-space and OECP measurements were performed utilizing the white set of foam core fiberglass sandwich materials, while the cavity measured another set of these same materials (the yellow one). Due to manufacturing, a tiny difference between the thickness of the samples of each set is present; this difference may be negligible for some tests, but, in this case, it affects the obtained permittivity. If the thickness of the samples is not accurately measured, errors will be introduced, especially in the case of the thinnest samples, like SCRAB 10. For example, errors on the order of 10% when measuring the thickness may incur an error of 10% in the extracted permittivity. As for the imaginary part of the permittivity, it is noticed that free-space does not have enough resolution to infer very low loss factors, mainly due to the error in phase introduced by the measurement setup, while OECP always returns higher values one more time.

Given that both the results from free-space and cavity agree with a variation of around ±10% in a frequency range of 8–13 GHz, it can be concluded that, for the frequency range of interest in this study (8–16 GHz), and for the specified materials under test, the results obtained with the free-space methodology are more reliable than the OECP results.

## 4. Conclusions

A complete methodology to estimate the complex permittivity of composite fiberglass materials utilizing the metal-backed free-space technique along with the PSO algorithm has been provided. The aim of the paper was to prove that for radiated-fields-like applications (radar cross-section (RCS), electromagnetic compatibility under high intensity radiated fields (EMC-HIRF)...) and for these kinds of aeronautical low-loss materials, the metal-backed free-space technique is reliable enough. To validate the results, the same materials have been measured with an OECP, but the agreement between the permittivity estimated by both techniques was not as expected. A further evaluation of two reference materials has shown that, due to the intrinsic inaccuracies of the OECP measurement procedure and the nature of the materials under test, the free-space approach returns more reliable results.

Bearing in mind the inhomogeneity of the considered materials, the proposed technique takes into account the whole sample, contrary to OECP, and is able to offer in just one measurement more reliable results for our purposes than other techniques presented (recall that OECP fails with material HiK500F). Another advantage of this methodology is the low manipulation required to prepare the test samples, which is particularly important for the special composition and fabrication process of the aeronautical composites.

To reinforce that metal-backed free-space is firm enough, an additional method has been applied, the split cavity resonator. The positive comparison of these last results have validated the free-space methodology explained along this paper for the extraction of permittivity, since the differences found in $\epsilon^{\prime }$ between cavity and free-space are mainly due to small deviations in material thickness. Nevertheless, further investigation is needed in free-space for the accurate extraction of the imaginary part of the permittivity. 

## Figures and Tables

**Table 1:** Materials under test. Dimensions.

Material Name	Height (mm)	Length (mm)	Thickness (mm)
SCRAB 4	200	200	6.3
SCRAB 9	200	200	4
SCRAB 10	200	200	2.3
SCRAB 13	200	200	9
Teflon	200	200	10
HiK500F	200	200	12.7

**Table 2:** SCRAB-II materials under test. Extracted mean permittivity (f = 8–16 GHz) for open-ended coaxial-probe (OECP) and free-space.

Material	Thickness (mm)	OECP-$\epsilon_{r}^{\prime }$	OECP-$\epsilon_{r}^{\prime \prime }$	Free-Space-$\epsilon_{r}^{\prime }$	Free-Space-$\epsilon_{r}^{\prime \prime }$
SCRAB 4	6.3	2.34	3.4$\times 10^{-2}$	1.58	3.6$\times 10^{-5}$
SCRAB 9	4	3.01	3.9$\times 10^{-2}$	2.10	9.4$\times 10^{-4}$
SCRAB 10	2.3	2.49	10.6$\times 10^{-2}$	2.23	2.0$\times 10^{-3}$
SCRAB 13	9	2.18	6.1$\times 10^{-2}$	1.35	3.0$\times 10^{-3}$

**Table 3:** Eccostock HiK500F. Extracted mean permittivity (f = 8–16 GHz) for OECP and free-space.

Material	Thickness (mm)	OECP-$\epsilon_{r}^{\prime }$	OECP-$\epsilon_{r}^{\prime \prime }$	Free-Space-$\epsilon_{r}^{\prime }$	Free-Space-$\epsilon_{r}^{\prime \prime }$
Eccostock HiK500F	12.7	22.89	2.0$\times 10^{0}$	31.83	5.0$\times 10^{-1}$

**Table 4:** SCRAB-II materials under test. Extracted mean permittivity (f = 8–13 GHz) for OECP, free-space and split cavity resonator.

Material	OECP-$\epsilon_{r}^{\prime }$	OECP-$\epsilon_{r}^{\prime \prime }$	Free-Space-$\epsilon_{r}^{\prime }$	Free-Space-$\epsilon_{r}^{\prime \prime }$	Cavity-$\epsilon_{r}^{\prime }$	Cavity-$\epsilon_{r}^{\prime \prime }$
SCRAB 4	2.34	4.7$\times 10^{-2}$	1.66	5.7$\times 10^{-5}$	1.77	1.3$\times 10^{-2}$
SCRAB 9	3.01	5.7$\times 10^{-2}$	2.15	9.6$\times 10^{-4}$	1.99	2.0$\times 10^{-2}$
SCRAB 10	2.51	10.6$\times 10^{-2}$	2.18	7.1$\times 10^{-4}$	1.70	2.0$\times 10^{-2}$
SCRAB 13	2.18	7.6$\times 10^{-2}$	1.33	2.5$\times 10^{-12}$	1.53	1.3$\times 10^{-2}$
